# Predicting existing targets for new drugs base on strategies for missing interactions

**DOI:** 10.1186/s12859-016-1118-2

**Published:** 2016-08-31

**Authors:** Jian-Yu Shi, Jia-Xin Li, Hui-Meng Lu

**Affiliations:** School of Life Sciences, Northwestern Polytechnical University, Xi’an, 710072 China

## Abstract

**Background:**

There has been paid more and more attention to supervised classification models in the area of predicting drug-target interactions (DTIs). However, in terms of classification, unavoidable missing DTIs in data would cause three issues which have not yet been addressed appropriately by former approaches. Directly labeled as negatives (non-DTIs), missing DTIs increase the confusion of positives (DTIs) and negatives, aggravate the imbalance between few positives and many negatives, and are usually discriminated as highly-scored false positives, which influence the existing measures sharply.

**Results:**

Under the framework of local classification model (LCM), this work focuses on the scenario of predicting how possibly a new drug interacts with known targets. To address the first two issues, two strategies, Spy and Super-target, are introduced accordingly and further integrated to form a two-layer LCM. In the bottom layer, Spy-based local classifiers for protein targets are built by positives, as well as reliable negatives identified among unlabeled drug-target pairs. In the top layer, regular local classifiers specific to super-targets are built with more positives generated by grouping similar targets and their interactions. Furthermore, to handle the third issue, an additional performance measure, Coverage, is presented for assessing DTI prediction. The experiments based on benchmark datasets are finally performed under five-fold cross validation of drugs to evaluate this approach. The main findings are concluded as follows. (1) Both two individual strategies and their combination are effective to missing DTIs, and the combination wins the best. (2) Having the advantages of less confusing decision boundary at the bottom layer and less biased decision boundary at the top layer, our two-layer LCM outperforms two former approaches. (3) Coverage is more robust to missing interactions than other measures and is able to evaluate how far one needs to go down the list of targets to cover all the proper targets of a drug.

**Conclusions:**

Proposing two strategies and one performance measure, this work has addressed the issues derived from missing interactions, which cause confusing and biased decision boundaries in classifiers, as well as the inappropriate measure of predicting performance, in the scenario of predicting interactions between new drugs and known targets.

## Background

It is a crucial step in drug discovery to identify targets (the druggable proteins related to diseases) for novel drug candidates [1, 2]. However, testing a large number of candidates in experiments would be still costly in both money and time. Based on the well-accepted assumption that similar drugs tend to interact with similar targets, computational methods (i.e. supervised machine learning) are able to predict novel drug–target interactions (DTIs) on a large scale by drug similarities and/or target similarities.

Generally, there are four scenarios of predicting interactions [3, 4] between (S1) known drugs and known targets; (S2) new drugs and known targets; (S3) known drugs and new targets; and (S4) new drugs and new targets. Here, a known (approved) drug is the drug having one or more known interactions with a targeted protein; a known target is the target interacting with one or more approved drugs; a new drug, referred to as a “drug candidate”, has no any known interaction; and a new target is the potential target having no known interaction with any drugs. It is remarkable that the appropriate cross validations for different scenarios should be adopted when assessing computational approach. Otherwise, over-optimistic results are perhaps obtained [4].

Since a set of known DTIs can be represented as a bipartite graph [5], network inference-based algorithms (e.g. [6]) are applied to predict new interactions by analyzing the topology of this graph. However, these algorithms cannot work well when the inference involves the drugs and/or the targets having no connection to the graph (e.g. in S2, S3 and S4). Matrix factorization algorithms have also been performed on the adjacent matrix of DTI graph to predict potential DTIs [7]. They suppose that a drug and a target may interact with each other if they share similar features in a common latent feature space (usually having fewer dimensions than the number of drugs and the number of targets). However, it is still hard to apply this approach in S2, S3, and S4, which are corresponding to the well-known cold-start problem. In addition, matrix factorization usually bears larger computational complexity.

Supervised classification models have been gained many concerns in DTI prediction [3, 4, 8–12], because they are able to handle all predicting scenarios and have the advantage of elucidating explicitly why a drug interacts with a target. Former supervised models can be approximately grouped into two categories: local classification model (LCM) and global classification model (GCM). LCM considers that the interactions between drugs and a focused target or between targets and a specific drug follow a common distribution [3, 8–10]. In contrast, GCM follows the assumption that DTIs crossing all drugs and all targets follow a common distribution [4, 10–12]. Usually, GCM needs much more memory space than LCM, because it always operates Kronecker product on both drug similarity matrix and target similarity matrix.

In common, supervised classification models regard existing DTIs as positive instances and unlabeled drug-target pairs (DTP) as negative instances respectively.

However, unlabelled DTPs include many unapproved DTPs as well as few potential DTIs which are not approved yet. In fact, few out of unlabelled DTPs are true DTIs but not collected when people extract DTI datasets. We call both potential DTIs and uncollected DTIs as missing DTIs in the context of DTI prediction because they have no technical difference.

From the point of view of supervised learning, a well-trained supervised classification model should have a decision boundary that separates positives and negatives significantly. In addition, an appropriate performance measure is crucial to fairly reflect the power of predicting models. Thus, missing DTIs would cause three important issues. Firstly, directly regarded as negatives, they induce a confusing decision boundary in the trained model, which usually cannot separate positives and negatives clearly. Secondly, they also cause a biased decision boundary in the classifier, by which positives tend to be determined as negatives. Thirdly, they are always discriminated as highly-scored false positives by predicting model, so as that the performance under existing measures is sensitive to missing interactions. Though some of the former approaches (e.g. [13, 14]) provided a start to address the first issue, they have not yet addressed the remaining two issues.

This work focuses on the prediction in scenario S2. To address the first two issues, we shall first introduce two strategies, Spy and Super-target, and further integrate them to form a two-layer local classification model. Then, to cope with the third issue, we shall present a new performance measure, Coverage, to assist the assessment of predicting model on the data containing missing DTIs. In addition, we shall demonstrate the superiority of our approach by comparing with two former approaches, one semi-supervised approach and one supervised approach, respectively.

## Methods

### DTI prediction as locally supervised classification

The interactions between *m* known drugs ({*d*_*i*_}, *i* = 1, …, *m*) and *n* known targets ({*t*_*j*_}, *j* = 1, …, *n*) could be organized by an interaction matrix *A*_*m* × *n*_, in which *a*(*i*, *j*) = 1 if there is a known interaction between drug *d*_*i*_ and target *t*_*j*_, and *a*(*i*, *j*) = 0 otherwise. In S2, given a new drug *d*_*x*_, the task is to predict the interaction between *d*_*x*_ and a known target *t*_*j*_ or determine how likely it interacts with *t*_*j*_. It can be also treated as a problem of regular supervised classification as follows: (1) labelling the known drugs *d*_*i*_ as a positive instance if *a*(*i*, *j*) = 1 (*d*_*i*_ interacts with *t*_*j*_), or as a negative instance (*d*_*i*_ doesn’t interact with *t*_*j*_); (2) training the classifier with the labels of {*d*_*i*_} and their pairwise similarities; (3) discriminating *d*_*x*_ as a positive or a negative instance in binary, or assigning it with a confidence score of being a positive instance by the trained classifier. The confidence score will be directly used to evaluate the performance of DTI prediction (Section Assessment).

The classifier used in our approach is Regularized Least Squared (RLS) classifier because its training only involves the solution of a linear system and its prediction at new testing samples is very elegant [15, 16]. Usually, for a testing sample *x* (corresponding to *d*_*x*_), its score of belonging to the *j*-th class is generated by the trained RLS classifier as follows,

1 $$ {f}_j(x)=\mathbf{K}\left(x,{\mathbf{X}}_{trn}\right){\left(\mathbf{K}\left({\mathbf{X}}_{trn},{\mathbf{X}}_{trn}\right)+\lambda \mathbf{I}\right)}^{-1}{\mathbf{Y}}_j, $$

where *f*_*j*_(*x*) is the predicted score of *x*, **K** is the kernel matrix directly derived from drug similarity matrix, the 1 × *m* matrix **K**(*x*, **X**_*trn*_) contains pairwise similarities between *d*_*x*_ and *m* known drugs, the *m* × *m* matrix **K**(**X**_*trn*_, **X**_*trn*_) contains pairwise similarities between *m* known drugs, *λ* is the regularization parameter (usually equal to 0.5), **I** is the *m* × *m* identity matrix and **Y**_*j*_ is the *m* × 1 class label vector of training samples corresponding to the *j*-th column of adjacent matrix (target *t*_*j*_). In the context of predicting interactions, *f*_*j*_(*x*) is just taken as *d*_*x*_’s confidence score *S*(*d*_*x*_, *t*_*j*_) of interacting with *t*_*j*_.

### Spy strategy for identifying reliable non-DTIs (negatives)

Since unlabeled drug-target pairs contain missing DTIs, simply regarding them as negative instances may cause a bad classifier (a bad predicting model) which has a confusing decision boundary between positives and negatives. In other words, it cannot separate newly-coming positives and negatives clearly. Aiming to recover those hidden positive instances in the unlabeled instances, we first utilized a semi-supervised strategy, *Spy* [17], to identify the reliable negatives (RN) from unlabeled instances. Since RN is significantly different to positives, a better classifier of less confusing boundary can be trained by positives (known DTI) and RN (reliable non-DTI).

**Fig. 1:**
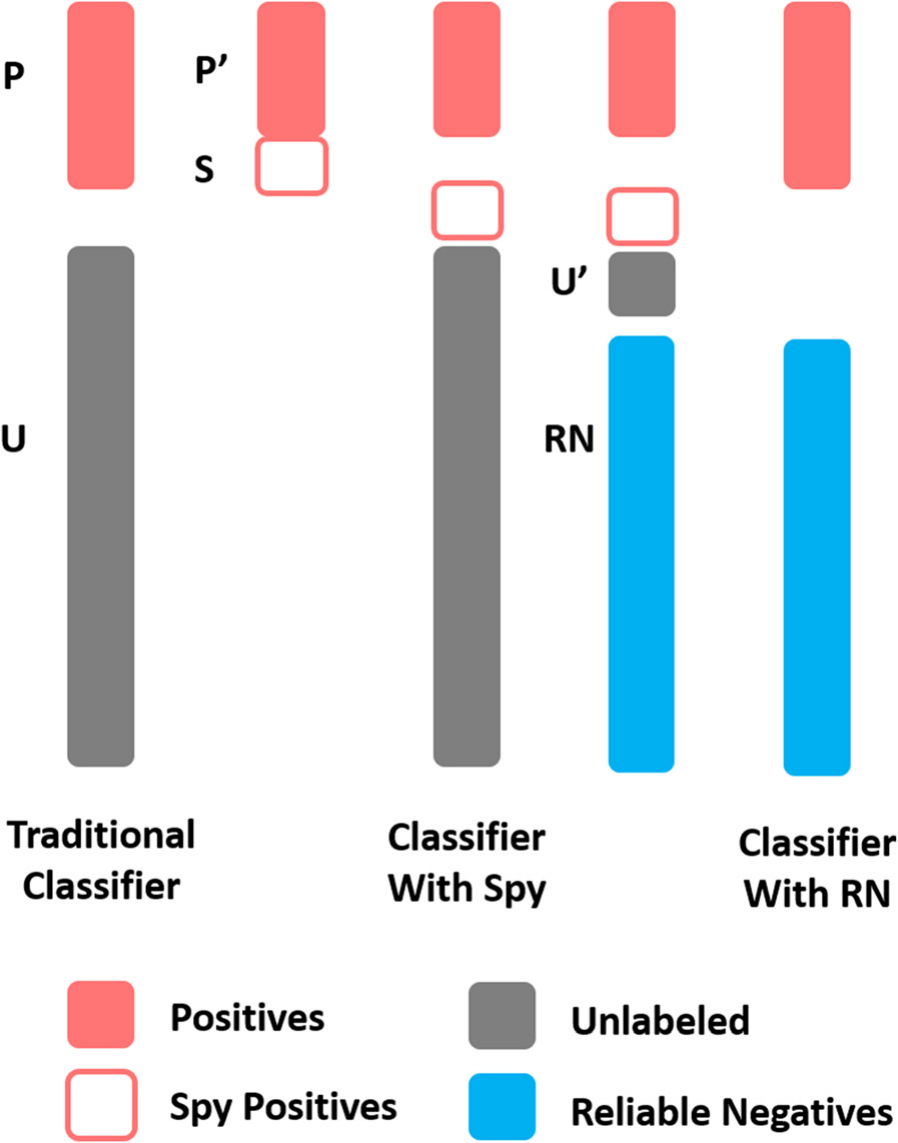
Spy strategy to identify reliable non-DTI. Positives (P), unlabeled instances (U), spy instances(S) taken from positives, and reliable negatives (RN) identified among unlabeled instances, are rendered with red bars, gray bars, red frames and blue bars respectively. The traditional classifier is trained by positives and unlabeled instances (treated as negatives directly). The Spy-based classifier is built by injecting S into U and investigating the behavior of S to identify RN. The final classifier is trained with positives and RN

Denote the set of labeled instances (known DTI) as *P* and all the unlabeled instances as *U*. A small set of labeled instances, *S*, are randomly selected from *P* and injected into *U*. Name the remaining labeled instances in *P* as *P*’ and the union set of *U* and *S* as *U*’ respectively. Behaving similarly to the unknown positive instances in *U,* the instances in *S* act as “spy” instances in *U*’. Therefore, they allow us to investigate the behavior of the unknown positive instances in *U* by the following steps: (1) the instances in *P*’ and *U*’ are labeled as positives and negatives respectively to build an ordinary classifier; (2) each instance in *U*’ is assigned with a predicting score of being a positive by this trained classifier; (3) the minimum of the scores of “spy” instances is taken as the threshold *t* to identify *RN*; (4) the instances in *U* having the scores less than t are determined as *RN*; (5) a new classifier is finally trained on *P* and *RN* to perform DTI prediction. Figure 1 illustrates this strategy.

Because the drug similarity matrix used in local classification model is unique, we assume that the thresholds of identifying RN in different local classifiers are identical. In practice, we estimated this unified threshold from the target which has the maximum number of drugs interacting with itself. The procedure is listed as follows: (1) randomly selecting 10 % “spy” instances out of *P* to determine the threshold, (2) repeating the selection ten times and obtaining ten thresholds, and (3) averaging the ten thresholds as the final threshold.

### Super-target strategy for grouping similar DTIs (positives) as many as possible

In DTI data, most of the targets may interact with only one or very few drugs. For example, 485 out of 664 targets in Enzyme dataset interact with less than 3 drugs (445 drugs in total), and 288 targets interact with only single drugs respectively [12]. In this case of instance imbalance, the training would build a classifier having a biased decision so as that the testing instances tend to be discriminated as negatives. Missing interactions aggravate the imbalance between few positives and many negatives.

**Fig. 2:**
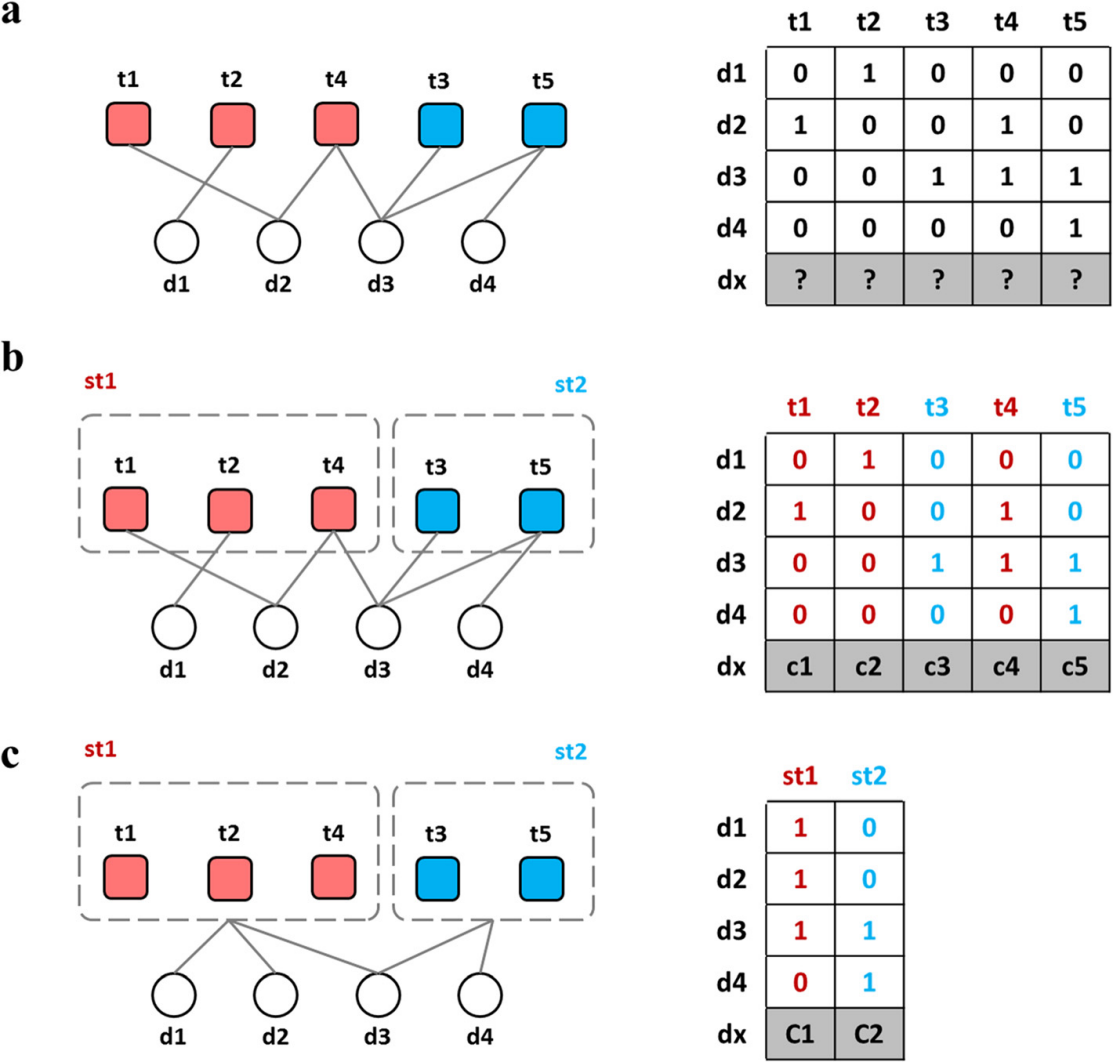
Super-target strategy to gather more similar DTIs (positives). (**a**) DTI graph and its adjacent matrix. (**b**) The groups of similar targets and their corresponding columns in the adjacent matrix. (**c**) Drug-supertarget interaction graph and its adjacent matrix. The graph (*left*) and the adjacency matrices (*right*) are different ways to represent the interactions between four drugs and five targets. The drugs (*circles*), targets (*squares*) and super-targets (*dashed rectangles*) are labeled by the names starting with “d”, “t” and “st” respectively. Similar targets are in the same color. The adjacency matrix between the drugs and super-targets (*bottom right*) is obtained from the original DTI matrix (*top right*) by performing the union operation on the columns corresponding to targets belonging in the same super-target

Inspired by compound screening, Super-target strategy is able to increase the number of positives by grouping similar targets and their interacting drugs [3]. A group of similar targets is named a *super-target* (Fig. 2). Super-target creates an additional layer of classifiers, which should further be incorporated into a regular local model to form a two-layer local model because our final goal is to determine how likely *d*_*x*_ interacts with *t*_*j*_. In this layer, a classifier of less biased boundary is trained under the condition of as many DTIs as possible. It is especially usefully in the case that no or only a few similar drugs interact with individual targets while more similar drugs interact with the super-target of those individual targets [3].

To predict how likely *d*_*x*_ interacts with *st*_*q*_, an ordinary classifier at the level of super-target is built by labeling all drugs linking to super-target *st*_*q*_ as positives and those not linking to it as negatives. Likewise, the confidence score *S*(*d*_*x*_, *st*_*q*_) of *d*_*x*_ interacting with *st*_*q*_ can be calculated by formula (1).

However, the original Super strategy in [3] did not consider that drugs interacting with all the target in a super-target are significantly different. The interactions between these drugs and a super-target are called the fake interactions of super-target, which may cause a bad classification boundary. We identified fake interactions by the following rule. If a drug interacting with a super-target ST, cannot find any of its top-K nearest neighbors (e.g. K = 3) among other drugs interacting with ST, its interaction with ST is marked as a fake. After checking all the super-targets with this rule, we removed the fake interactions to obtain the cleaned super-targets which group similar positives as many as possible.

### Two-layer local classification model

We integrated two strategies for missing interactions to form a two-layer local classification model. For target *t*_*j*_, in the top layer, the model clusters similar targets into super-targets and predicts how likely *d*_*x*_ interacts with a super-target *st*_*q*_ which contains *t*_*j*_ by a traditional supervised learning. Then, in the bottom layer, it predicts how likely *d*_*x*_ interacts with *t*_*j*_ ∈ *st*_*q*_ via the Spy-based classifier built by positives and RN. The final confidence score of *d*_*x*_ interacting with *t*_*j*_ is calculated by

2 $$ S\left({d}_x,{t}_j\right)=\sqrt{S\left({d}_x,s{t}_q\right)S\left({d}_x,{t}_j\in s{t}_q\right)} $$

Because some super-targets may contain fewer similar positives and more dissimilar positives, an adaptive rule was designed to determine when to apply Super-target: (1) for each drug interacting with *st*_*q*_, counting the occurring number *n*_*u*_^*q*^ of its *K* nearest neighbors (e.g. K = 3) among other drugs interacting with *st*_*q*_; (2) for all the drugs interacting with *st*_*q*_, averaging the occurring numbers by *ñ*_*q*_ = ∑_*u* = 1_^*U*^*n*_*u*_^*q*^/*U*, where *U* is the number of drugs interacting with *st*_*q*_; (3) rejecting the Super-target strategy if *ñ*_*q*_ < *K*/2, otherwise accepting it.

Such combination has the following advantages. Super-target enables the training under the condition of as many similar DTIs as possible, so as to relax the instance imbalance and generate the less biased decision boundary. Meanwhile, training with positives and reliable non-DTIs, Spy strategy guarantees a less confusing decision boundary between DTIs and non-DTIs.

### Assessment

The validation for predicting interactions between known targets and new drugs should be appropriately designed [4], otherwise the prediction is over-optimistic (e.g. [9] and [13]). We followed the five-fold cross validation (5-CV) in [3, 4, 14] to evaluate the proposed approach. The drugs in a given dataset are randomly split into 5 non-overlapping subsets of approximately equal sizes. Out of the five subsets, one is used as the testing set and the remaining subsets are taken as the training set. This process is then repeated five times, to use each of the five subsets as the testing set in turn.

In each round of CV, the predicting performance is usually measured by *the Area under the Receiver Operating Characteristic Curve* (AUC). The corresponding average of AUC in five rounds is taken as the final indicator of assessment. In practices, AUC is defined as

3 $$ AUC=\left({n}^{\prime }+0.5{n}^{{\prime\prime}}\right)/\left({n}^{\prime }+{n}^{{\prime\prime}}\right), $$

where *n*′ and *n*″ are the numbers of the cases that positives are larger than and equal to negatives in terms of predicting scores respectively. The larger, the better. Deriving from the traditional supervised classification, the calculation of AUC has the need to compare all positives with all negatives. AUC reflects how often positives are greater than negatives on the average in terms of the scores assigned by the predicting model.

Like compound or target screening, computational approaches predict potential interactions (positives) from unlabeled drug-target pairs by selecting the instances of high scores as the candidates. Thus, missing DTIs in the testing negative instances would be recognized as potential interactions if they are assigned with high scores (lower ranks). However, they are usually counted as false positives in assessment since they are simply labeled as negatives. Unfortunately, the calculation of AUC cannot reflect the situation. In consequence, there is a need for another performance measure to assist the assessment.

A new measuring index, *Coverage*, is presented to additionally assess the prediction on the data containing missing interactions. It is defined as follows

4 $$ Coverage=\frac{1}{p}{\displaystyle \sum_{i=1}^p\underset{t\in {T}_i}{ \max}\left\{ rank\left({d}_i,t\right)\right\}}-1, $$

where *p* is the number of the testing drugs and *T*_*i*_ denotes the set of the targets interacting with *d*_*i*_. The smaller, the better. When predicting interactions for a new drug, *Coverage* is able to evaluate how far, on the average, we need to go down the target list in order to cover all the proper targets of the drug.

**Fig. 3:**
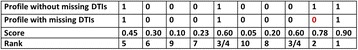
A toy case showing the difference between AUC, AUPR and Coverage. The first row denotes an interaction profile between a drug and 10 targets. The second row denotes an interaction profile with one missing interaction by removing the 9-th interaction from the first row. The third row contains the predicted scores generated by performing a predicting approach on the second row. The last row lists the ranks corresponding to the predicted scores. The values of AUC, AUPR and Coverage accounting for the first row, are 0.833, 0.683 and 4 respectively. In contrast, after labelling the missing interaction as a positive correctly, those value of AUC and AUPR (corresponding to the first row) would change to 0.938, 0.912, but the value of Coverage doesn’t change. AUPR is significantly sensitive to the missing interaction, AUC is moderately sensitive and Coverage is the most robust

We believe that the number of unlabeled positives (missing data) is small, and most of them are assigned with the scores higher than the lowest scores of known DTIs. In other words, Coverage can embrace as many missing interactions as possible even though they are treated as negatives during the assessment. Therefore, Coverage is more robust than AUC when missing interactions occur. A toy example of calculating the values of AUC and Coverage is shown in Fig. 3. Totally, a good predicting approach is able to generate high AUC as well as low Coverage.

## Results and discussion

### Data

The adopted datasets in our experiments were originally collected by [12] and further used in subsequent works [3, 4, 6–11, 13, 14] as the benchmark. All the DTIs in the original work were split into four subsets in terms of the type of protein targets, including enzyme, ion channel, GPCR and nuclear receptor. Here, the four DTI datasets are shortly denoted as EN, IC, GPCR and NR respectively. Former publications generally used chemical-structure-based drug similarities and sequence-based target similarities respectively when predicting DTIs [3, 12]. The pairwise drug similarity was measured by aligning the chemical structures of two drugs [18]. The pairwise target similarity was derived from by Smith-Waterman alignment [19]. More details can be found in the original work [12]. Because the drugs having different structures may interact with common targets and the proteins having different sequences may be targeted by common drugs, we also adopted additional non-structure-based drug similarity and non-sequence-based target similarity which were proposed in [3]. The new pairwise drug similarity was calculated by comparing the class labels of two drugs according to *Anatomical Therapeutic Chemical* (ATC) *Classification System*. The new pairwise target similarity was calculated by comparing the functional categories of two targets according to the annotation of *HUGO Gene Nomenclature Committee* (http://www.genenames.org/). The details of similarity calculation can be obtained in [3]. The final drug/target similarity matrix we used was just the average of the new similarity matrix and the previous similarity matrix. Based on DTI adjacent matrix, drug similarity matrix was used to train classifiers and target similarity matrix was used to form super-targets. The datasets used in the following experiments can be downloaded from the web address provided in [3, 12].

### The effectiveness of individual strategies and their combination

To validate the effectiveness of two proposed strategies and their combination, we first run the ordinary local model (RLS); then run the model incorporated with Spy strategy alone (RLSm_spy); after that, run the model extended by only Super-target strategy (RLSm_super); last, combined two strategies and run the model again (RLSm_comb). The performance was measured by both AUC and Coverage (Table 1). Compared with RLS, RLSm_spy is better because of the less confusing boundary generated by positives and reliable-negatives at the level of targets. RLSm_super outperforms RLS as well because of the less biased boundary generated by grouping similar DTIs as many as possible at the level of super-targets. As expected, RLSm_comb wins the best among above all approaches and improves the prediction significantly (compared with RLS). These results demonstrate the effectiveness of our strategies for missing interactions.

**Table 1:** Comparing local model with the models having Spy and Purified Super-target strategies respectively

	RLS		RLSm-spy		RLSm-super		RLSm-comb	
	AUC	Coverage	AUC	Coverage	AUC	Coverage	AUC	Coverage
EN	0.844	129.596	0.847	123.966	0.848	121.769	**0.853**	**119.265**
IC	0.843	50.186	0.844	45.824	0.844	45.671	**0.847**	**43.162**
GPCR	0.856	18.752	0.869	16.850	0.890	16.216	**0.895**	**14.423**
NR	0.878	4.846	0.877	4.900	**0.889**	**4.000**	**0.889**	4.073

The bold entries denote the best results on the benchmark datasets

### Comparison with other approaches

Furthermore, our approach was compared with two recent approaches, NetCBP [14] and KronRLS [4] (Table 2). Both of them adopted the same 5-CV to validate the prediction. NetCBP, a semi-supervised approach, only used AUC to measure its performance, while KronRLS, an ordinary supervised approach, used AUC as well as AUPR (*the Area under Precision-Recall Curve*) to measure its performance. Their results were generated by only using chemical-structure-based drug similarity and sequence-based target similarity [4, 14]. To make a fair comparison with these approaches, using exactly same similarity matrices, we run the proposed two-layer local model and assessed it by both AUC and AUPR (the results denoted as RLSm-comb-less). In addition, we listed the results obtained by combined similarity matrices (Section Data) together. The comparison shows that our approach is superior to these two approaches (Table 2).

**Table 2:** Comparison with other approaches

	EN	IC	GPCR	NR
	AUC|AUPR	AUC|AUPR	AUC|AUPR	AUC|AUPR
NetCBP	0.825|-	0.803|-	0.824|-	0.839|-
KronRLS	**0.837**|0.361	0.802|0.258	0.852|0.378	0.846|0.493
RLSm-comb-less	0.816|**0.381**	**0.805**|**0.386**	**0.858|0.459**	**0.871**|**0.496**
RLSm-comb	**0.853|0.432**	**0.847|0.430**	**0.895|0.487**	**0.889|0.549**

RLSm-comb-less is our approach running with chemical-structure-based drug similarity and sequence-based target similarity. The bold entries denote the best results in terms of both AUC and AUPR

### Analysis on AUC, AUPR, and coverage

Different performance measures should be applied in appropriate cases. In a supervised classification problem, when the number of negatives greatly exceeds the number of positives, AUC is an optimistic measure [20]. Such instance imbalance in DTI prediction is significant. In this case, AUPR is more appropriate than AUC since it performs great penalty on highly-scored false positive predictions [20]. However, such penalty may cause pessimistic results because those highly- scored false positives are possibly unlabeled positives mixed in negatives.

A toy example in Fig. 3 illustrates how missing interactions influence the values of AUC, AUPR, and Coverage. Suppose that the values of AUC (0.833), AUPR (0.683) and Coverage (4.000) are calculated respectively when the interaction between the drug and the ninth target is missing. Remarkably, when the missing interaction is found back, the corresponding values are 0.938, 0.912 and 4.000 accordingly. Obviously, AUPR is quite sensitive to missing interactions assigned with high predicting scores since AUPR’s value changes sharply. In contrast, AUC is moderately sensitive and Coverage is the most robust (it doesn’t change in this case). This is the reason why we didn’t use AUPR but use both AUC and Coverage to assess the prediction when investigating the strategies for missing. When assessing the performance of a predicting approach, one should keep above points in mind. The further analysis about Coverage is depicted in the next section.

### Message from coverage

To elucidate the message behind Coverage, we compared our approach with two boundary-line approaches, Random prediction, and Oracle prediction. The former randomly assigns the confidence scores to all drug-target pairs, while the latter supposes both known interactions and unlabeled pairs are perfectly labeled with 1’s and 0’s respectively. Since the values of AUC generated by these two boundary-line approaches definitely ~0.5 and 1.0 respectively, we only focused on their values of Coverage, which denoted as *C*_*random*_ and *C*_*oracle*_ respectively. These two values were further used to normalize the Coverage value of the proposed approach into [0, 1]. The normalized Coverage is defined as *NC* = (*Coverage* − *C*_*oracle*_)/(*C*_*random*_ − *C*_*oracle*_). The smaller, the better. In addition, we calculated the ratio (*C*/# *T*) between Coverage and the number of targets as well. Both of NC and this ratio facilitate the comparison across different datasets of varied sizes (Table 3).

**Table 3:** Message from Coverage

	C_random_	C_oracle_	Coverage	NC	#T	C/#T
EN	437.076	5.575	119.265	0.263	664	0.180
IC	147.557	6.029	43.162	0.262	204	0.212
GPCR	61.148	1.848	14.423	0.212	95	0.152
NR	14.185	1.667	4.073	0.192	26	0.157

#T is the number of targets in dataset, C/#T is the ratio between Coverage and #T, C_random_ is the Coverage derived from random prediction, and C_oracle_.is the Coverage corresponding to oracle prediction and NC is the normalized Coverage

Three messages can be observed from the results in Table 3. (1) RLSm-comb is significantly better than Random prediction in terms of Coverage value. (2) The significant difference between RLSm-comb and Oracle prediction highlights the needs to extract better drug similarities and to develop better models for DTI prediction. (3) Most importantly, related to the cost of screening, both *NC* and *C*/# *T* reflect that ~20 % out of all the targets along the candidate list, on the average, should be checked to cover all the proper targets interacting with the drug. Therefore, Coverage is able to indicate the predicting performance more informatively than AUC or AUPR.

## Conclusions

In this paper, when predicting potential targets for new drugs under the framework of local classification model, we have addressed three important issues caused by missing DTIs. First, simply treating directly unlabeled instances as negatives would cause the confusing decision boundary between positives and negatives. To cope with it, we have adopted a semi-supervised strategy, Spy, which can identify reliable non-DTIs (negatives) from unlabeled DTP (unlabeled instances) by investigating the behavior of DTI (positives) among unlabeled DTP. Thus, Spy enables the training to be under the condition of positives and reliable negatives, so as to generate a less biased decision boundary.

Secondly, directly aggravating the toughness of few positives, missing DTIs also cause a biased decision boundary which tends to predict newly-coming positives as negatives. To address it, we have adopted the strategy, Super-target, to cluster similar targets as well as the drugs interacting with them. Super-target creates an additional layer of the Spy-based local classification model. In this layer, a classifier of less biased boundary is trained under the condition of as many DTIs as possible. According to the number of similar drugs interacting with a super-target, we have also introduced the adaptive combination of two strategies to form a two-layer predicting model.

Thirdly, existing measures (e.g. AUC and AUPR) of predicting performance do not consider missing interactions, which are always assigned with high scores by predicting models and counted as false positives in assessment. As a complementary, we presented Coverage which is robust to highly-scored missing interactions. Besides, it enables us to evaluate how far we need to walk along the list of targets in order to visit all the proper targets of the queried drug.

In short, having less confusing and less biased decision boundaries at the levels of target and super-target respectively, the proposed two-layer model first predicts how possibly a new drug interacts with a super-target, then predicts how possibly it interacts with a member target contained in that super-target, and its performance is assessed by the new measure (Coverage), in addition to the traditional measure (AUC).

Finally, based on four real benchmark datasets, we have demonstrated that our approach is able to not only cope with missing interactions but also perform superiorly to two other approaches with respect to the problem of predicting interactions between known targets and new drugs. Moreover, our approach can be applied to the symmetric predicting scenario S3 as well.

Nevertheless, we are still walking on a long road. The performance of DTI prediction, especially measured by Coverage, reminds us that predicting potential targets for new drugs still remains a tough challenge. More efforts on drug similarity metric, as well as the predicting model and the appropriate assessment for missing interactions, should be done.

## Abbreviations

5-CV, five-fold cross validation; ATC, Anatomical Therapeutic Chemical; AUC, the area under the receiver operating characteristic curve; AUPR, the area under precision-recall curve; DTI, drug-target interaction; DTP, drug-target pair; RLS, Regularized Least Squared; GCM, global classification model; LCM, local classification modelv; RN, reliable negative.
